# Prediction of IDH1 gene mutation by a nomogram based on multiparametric and multiregional MR images

**DOI:** 10.1016/j.clinsp.2023.100238

**Published:** 2023-06-22

**Authors:** Jinjing Zheng, Haibo Dong, Ming Li, Xueyao Lin, Chaochao Wang

**Affiliations:** Department of Radiology, Ningbo Medical Center Lihuili Hospital, Ningbo University, China

**Keywords:** Glioma of the brain, Magnetic resonance imaging, Isocitrate dehydrogenase, Radiomics, Genotype

## Abstract

**Objective:**

To investigate the value of a nomogram based on multiparametric and multiregional MR images to predict Isocitrate Dehydrogenase-1 (IDH1) gene mutations in glioma.

**Data and methods:**

The authors performed a retrospective analysis of 110 MR images of surgically confirmed pathological gliomas; 33 patients with IDH1 gene Mutation (IDH1-M) and 77 patients with Wild-type IDH1 (IDH1-W) were divided into training and validation sets in a 7:3 ratio. The clinical features were statistically analyzed using SPSS and R software. Three glioma regions (rCET, rE, rNEC) were outlined using ITK-SNAP software and projected to four conventional sequences (T1, T2, Flair, T1C) for feature extraction using AI-Kit software. The extracted features were screened using R software. A logistic regression model was established, and a nomogram was generated using the selected clinical features. Eight models were developed based on different sequences and ROIs, and Receiver Operating Characteristic (ROC) curves were used to evaluate the predictive efficacy. Decision curve analysis was performed to assess the clinical usefulness.

**Results:**

Age was selected with Radscore to construct the nomogram. The Model 1 AUC values based on four sequences and three ROIs were the highest in these models, at 0.93 and 0.89, respectively. Decision curve analysis indicated that the net benefit of model 1 was higher than that of the other models for most Pt-values.

**Conclusion:**

A nomogram based on multiparametric and multiregional MR images can predict the mutation status of the IDH1 gene accurately.

## Highlights

• Combination of clinical factors and radiomic features to construct models for predicting IDH1 gene mutations in glioma.
• Multiparametric and multiregional MR images were used to achieve more accurate prediction performance.
• The model performance with different regions of interest for different sequences was compared.

## Introduction

Gliomas are the most common primary malignant tumors of the brain. In 2016, molecular typing was added to the World Health Organization classification of central nervous system tumors, and the 5^th^ edition in 2021 further emphasized the importance of genetic and molecular changes in the characterization of central nervous system tumors.^1^^,^^2^ Isocitrate dehydrogenase-1 is an essential molecular biomarker for glioma and is expressed in two states: Wild-type (IDH1-W) and Mutant (IDH1-M). Mutations in the IDH1 gene were the first detectable genetic alterations in glioblastomas, and the mutated IDH protein is thought to have a relatively good prognosis by competitively inhibiting participation in histone and DNA demethylation, thereby blocking cell differentiation and reducing tumor cell proliferation.^3^, ^4^, ^5^ Accurate prediction of glioma IDH1 mutation before treatment is of great significance to guide individualized treatment and prognosis assessment, which has become a research hotspot in radiogenomics in recent years.^6^ Since MR images contain information related to the pathophysiology of tumors, radiomics quantitatively analyzes image information, and high-throughput extracts with minable high-dimensional features that provide additional information about the microvasculature and microstructure of tumors which can be used to build statistical models.^7^, ^8^, ^9^ In previous studies that mostly involved individual sequences or individual regions of interest, the characteristics of tumor heterogeneity were not fully and completely reflected, and the accuracy needed to be improved.^10^, ^11^, ^12^ In this study, the region of Contrast-Enhanced Tumor (rCET), the region of Necrosis (rNec), and the region of Edematous tumor (rE) were outlined separately based on four MRI conventional sequences (T1, T2, FLAIR, T1C) and the clinical features combined with nomogram signatures were used to construct prediction models. A nomogram is a tool that allows for quantitative and visual convergence of radiomic features and clinical factors.^13^^,^^14^ It assigns a corresponding value to each risk factor and generates a visual probability estimate that ultimately helps clinicians predict patient prognosis.^15^, ^16^, ^17^ The authors aimed to develop the most clinically useful nomogram to accurately distinguish the mutational status of IDH1.

## Materials and methods

### Patient selection

The Ethics Committee of the hospital gave its approval to this retrospective investigation, and they waived the requirement of obtaining consent. The Ethics Committee study protocol number is KY2022PJ184. The study has been carried out in accordance with the STROBE Statement. Patients who met the following criteria from February 2016 to June 2022 were collected for inclusion in this study: 1) Confirmed to have glioma by postoperative immunohistochemistry; 2) Clinical features and IDH1 immunohistochemistry results; 3) MR images performed preoperatively with no obvious artifacts, including T1-weighted, T1-weighted gadolinium contrast-enhanced, T2-weighted, and T2-weighted FLAIR sequences (short for T1, T1C, T2, and FLAIR), which were available before treatment. The exclusion criteria were as follows: 1) A history of surgery or chemoradiation therapy; 2) Hemorrhage or calcification in the tumor or a tumor without necrotic areas or with weak enhancement. A total of 110 patients with glioma were enrolled, of whom 62 were male and 48 were female; 33 cases involved IDH1 mutant and 77 IDH1 wild type. The patients were randomly divided into a training group (77 cases, 21 IDH1-M type, 56 IDH1-W type) and a validation group (33 cases, 12 IDH1-M type, 21 IDH1-W type) according to a 7:3 ratio.

### Data Acquisition of MRI

All preoperative MRIs were performed on a 3.0 T MRI scanner (Discovery, GE Healthcare, Milwaukee, WI, USA) with an eight-channel head coil. 1) The conventional MRI scan sequence included the following: a T1-weighted sequence (T1): TR1750 ms, TE24 ms, TI780 ms; a T2-weighted sequence (T2): TR6240 ms, TE94 ms; a liquid attenuated inversion recovery T2 sequence (FLAIR): TR8400 ms, TE150 ms matrix 288×224, FOV 240×240 mm, layer spacing 1 mm, layer thickness 4 mm, 24 scanned layers; 2) A TIWI-enhanced MRI sequence (T1C): a cross-sectional T1WI sequence was scanned after injection of the contrast agent gadodiamide (GE Pharmaceuticals) at 0.2 mL/kg, with the same parameters as the flat-scan TIWI sequence.

### Selection of clinical characteristics

The clinical characteristics included sex, age, and seven tumor morphological features, including midline deviation (yes or no), growth across the midline (yes or no), tumor location (1 refers to left, right or bilateral hemispheres; 2 refers to frontal, occipital, parietal, temporal or other lobes), degree of enhancement (mild, obvious enhancement), border (clear or indistinct), and tumor size (maximum cross-sectional diameter of the tumor, in mm units). Using SPSS 25.0 software, continuous variables were first tested by the Kolmogorov-Smirnov test to evaluate whether the data conformed to a normal distribution. Categorical data that were normally distributed are expressed as the mean ± standard deviation, based on an independent samples *t*-test or ANOVA; those not satisfying this criterion are expressed as median or quartiles, as based on the Mann-Whitney *U* test. Categorical variables were expressed as frequencies, and the Chi-Square test or Fisher's exact test was used. Clinical features were screened in the training set, and univariate analysis was conducted to identify clinical features that were significantly different between the IDH1-M and IDH1-W groups in the training and validation sets. Multivariate logistic regression analysis was then performed on the training set to examine it for independent predictors.

### Pre-processing, segmentation, and feature extraction of images

GE AI-Kit (Artificial Intelligence Kit, GE Healthcare, Chicago, IL, USA; Version: 3.3) software was applied to perform image pre-processing, such as coregistration using T1C images as a template, denoising, intensity normalization, skull stripe, bias correction, and image resampling to 1×1×1 mm isotropic voxels with linear interpolation.

The T1C images were outlined layer by layer by two neuroimaging physicians on the open-source software ITK-SNAP (Version 3.4.0, http://www.itksnap.org/) and reviewed by one senior neuroimaging specialist to select the outlined ROI (region of interest, ROI). For each patient, other MRI sequences were co-aligned with reference to T1C. Three tumor ROIs were segmented, including the Contrast-Enhanced area (rCET), Necrosis area (rNec), and Edema area (rE). The edema area may include both peritumoral edema and any non-enhancing tumor, so multiple sequences were compared to ensure the accuracy of the ROI. Then, these three regional contours were mapped to each patient's MRI sequence and used for feature extraction.

The AI-Kit software performed image feature extraction for the MR images, and 396 radiomics features were calculated from each ROI, including intensity, morpho-logic, histogram, and textural parameters such as Gray-Level Co-occurrence Matrix (GLCM), Gray-Level Size Zone Matrix (GLSZM), Neighboring Gray Tone Difference Matrix (NGTDM), and Gray-Level Run Length Matrix (GLRLM).

The above data were pre-processed, and all data were divided into training and validation groups at a ratio of 7:3. Feature selection and model construction was completed in R software (v.4.2.1; http://www.Rproject.org). The minimum Redundancy Maximal Relevance (mRMR) algorithm was performed using the “mRMRe” package. Least Absolute Shrinkage and Selection Operator (LASSO) analysis was used to select the non-zero coefficient features that were applied to the model for predicting IDH1 gene expression types, and these features were combined with non-zero coefficients to construct a formula that was employed to calculate Radscore for each glioma case. The Wilcoxon test was utilized to compare the differences in Radscores across IDH1 gene status. The interobserver agreement between two neuroradiologists on visual features was assessed by the Intragroup Correlation Coefficient (ICC) in 30 randomly chosen patients. ICC scores greater than 0.85 was considered satisfactory for the protocol.

### Construction and assessment of predictive models

Radscore and the selected clinical predictors were included in the multivariate logistic regression model using R software, and the combined predictive models for predicting IDH1 gene expression types (i.e., the radiomics nomogram) were also established. The diagnostic efficacy of the eight prediction models was evaluated by implementing ROC curves using the following criteria: the diagnostic efficacy was low when 0.5 ≤ AUC < 0.7, moderate when 0.7 ≤ AUC < 0.9, and high when AUC ≥ 0.9. Calibration curves and the Hosmer-Lemeshow test were employed to estimate the concordance between the predicted probabilities of the model and the actual results. The model demonstrated excellent prediction if the calibration curves had a good fit and a statistical significance of p > 0.05. The authors set up eight prediction models based on different sequences or different regions of interest. Model 1 represented the combined model of all sequences and all ROIs, models 2‒5 were based on T1, T2, FLAIR, and T1C, and models 6‒8 were based on rCET, rNec, and rE, respectively. Each prediction model incorporated the screened clinical features. Decision curves were applied to appraise the net benefit of the clinical decision and to compare the clinical value of various prediction models. Predictive models provided clinical consequences on the threshold probability basis from which the net benefits were derived. All models were compared based on ROC and decision curve analyses, from which the optimal model was selected.

## Results

### Clinical characteristics of the patients

Based on the data in the last column of Table 1, the p-values for all characteristics are greater than 0.05, there were no significant differences in IDH1 gene status, WHO classification status, or clinical characteristics between the training and validation groups (Table 1). According to univariate logistic regression analysis, there was a significant difference in age (p < 0.001) between the IDH1-M and IDH1-W groups in the training set. Features with p ≥ 0.05 were excluded. After multivariate logistic regression analysis, age (p < 0.001) was still selected to construct the predictive model.

**Table 1:** Clinical and morphological features of patients in the training and validation groups.

Variables		Training group (n = 77)		p	Validation group (n = 33)		p	p
		IDH-M (n = 21)	IDH-W (n = 56)		IDH-M (n = 12)	IDH-W (n = 21)		
Grade^a^	2	1	1		1	0		
	3	7	4		3	3		
	4	13	51	*	8	18	*	**0.296**
Gender	Male	9 (42.9)	32 (57.1)		14 (66.7)	7 (58.3)		
	Female	12 (57.1)	24 (42.9)	**0.388**	7 (33.3)	5 (41.7)	**0.918**	**0.425**
Age (years)	Age<35	3 (14.3)	7 (12.5)		0 (0.0)	1 (8.3)		
	36 ≤ Age < 50	14 (66.7)	6 (10.7)		3 (14.3)	3 (25.0)		
	51 ≤ Age < 65	4 (19.0)	24 (42.9)		12 (57.1)	6 (50.0)		
	Age > 66	0 (0.0)	19 (33.9)	**<0.001**	6 (28.6)	2 (16.7)	**0.432**	**0.194**
Midline shift	No	3 (14.3)	21 (37.5)		7 (33.3)	3 (25.0)		
	Yes	18 (85.7)	35 (62.5)	**0.092**	14 (66.7)	9 (75.0)	**0.914**	**0.928**
Cross midline growth	No	20 (95.2)	47 (83.9)		18 (85.7)	11 (91.7)		
	Yes	1 (4.8)	47 (83.9)	**0.350**	3 (14.3)	1 (8.3)	**1.000**	**0.901**
Tumorlocation^a^	Left hemisphere	8 (38.1)	30 (53.6)		7 (33.3)	2 (16.7)		
	Right hemisphere	12 (57.1)	20 (35.7)		11 (52.4)	9 (75.0)		
	Bilateral	1 (4.8)	6 (10.7)	**0.220**	3 (14.3)	1 (8.3)	**0.439**	**0.099**
Tumorlocation^2^	Frontal lobe	12 (57.1)	20 (35.7)		7 (33.3)	5 (41.7)		
	Occipital lobe	1 (4.8)	6 (10.7)		1 (4.8)	0 (0.0)		
	Parietal lobe	3 (14.3)	5 (8.9)		3 (14.3)	0 (0.0)		
	Temporal lobe and others	5 (23.8)	25 (44.6)	**0.220**	10 (47.6)	7 (58.3)	**0.457**	**0.529**
Enhancement degree	Mild	5 (23.8)	4 (7.1)		5 (23.8)	2 (16.7)		
	Obvious	16 (76.2)	52 (92.9)	**0.103**	16 (76.2)	10 (83.3)	**0.968**	**0.316**
Border	Clear	4 (19.0)	8 (14.3)		5 (23.8)	3 (25.0)		
	Indistinct	17 (81.0)	48 (85.7)	**0.873**	16 (76.2)	3 (25.0)	**1.000**	**0.418**
Tumor size (mm)	Mean (SD)	44.5 (13.1)	45.5 (16.6)	**0.797**	44.5 (15.3)	40.6 (13)	**0.461**	**0.507**
Radscore	Median [iqr]	0.4 [0.0, 1.8]	-2 [-2.6, -1.6]	<0.001	44.5 (15.3)	0.5 [-0.3, 0.6]	<0.001	0.508

p < 0.05 was statistically significant.

a Grade is not used as a clinical feature for selection.

### Selection of radiomic features

**Figure 1:**
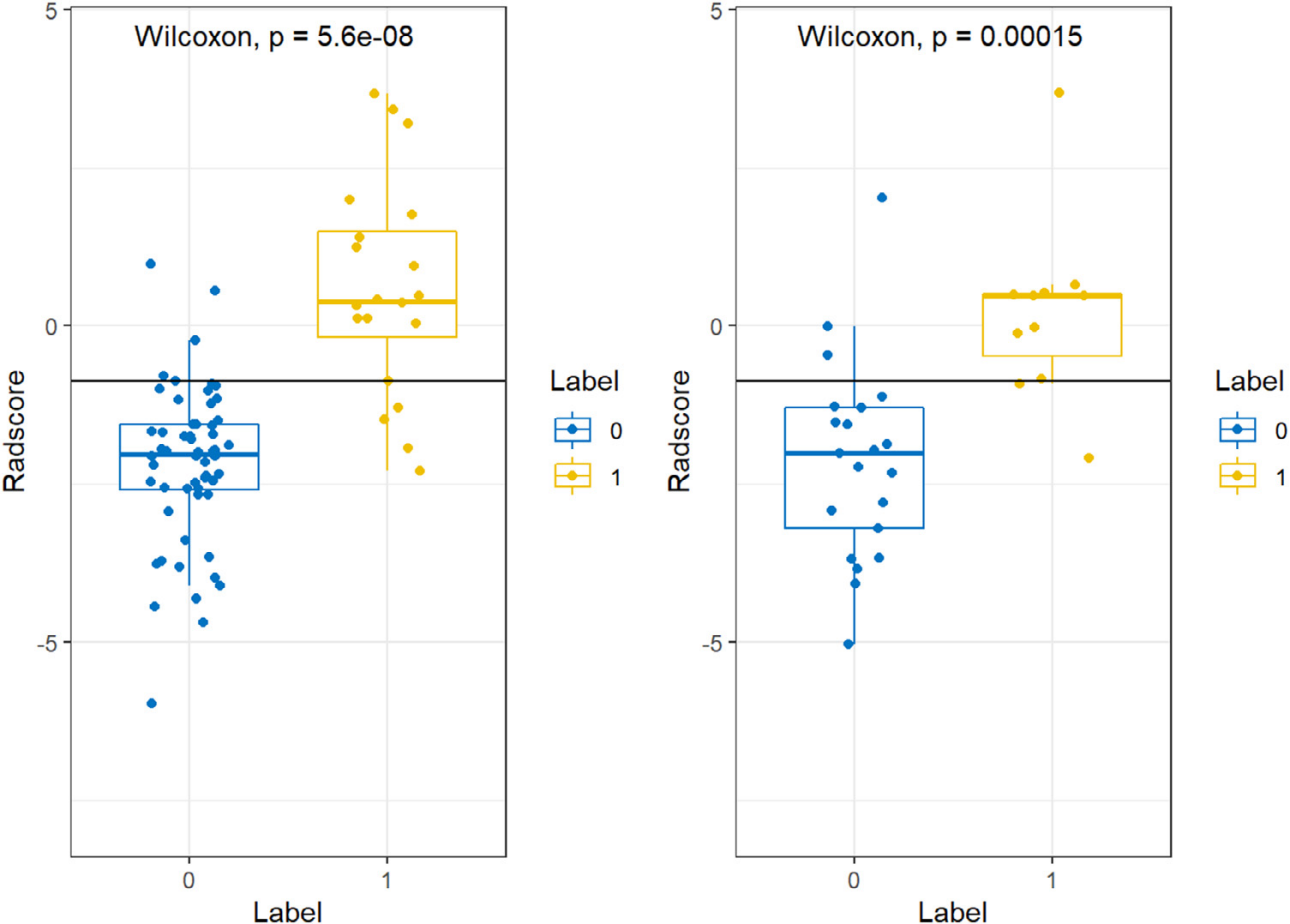
Comparison of the Radscore box plots under different labels for Model 1. The left side is the training cohort, and the right side is the validation cohort, and the p-values are less than 0.05, indicating that Radscore in the two cohorts were different under different labels.

The minimum Redundancy Maximal Relevance (mRMR) algorithm was used to extract the most robust features in the training set from 4752 valid radiomic features in model 1, and then nonzero coefficients chosen by LASSO as the optimal 7 features were utilized to establish the radiomics model and Radscore formula (Table 2). The Radscore was calculated for each patient in the training and validation group, and the results showed that the Radsore of IDH1-M was lower than that of IDH1-W, and the difference was statistically significant, as shown in the diagram in the box figure (Fig. 1). Models 2‒5 were all constructed based on 1189 radiomic features, and 9, 4, 6 and 6 non-zero coefficient features were extracted, respectively (Table 2). The total number of radiomic features for models 6‒8 was 1595, and 6, 3 and 5, respectively (Table 2). The 4,752 radiomic features extracted from these multiple-segmented subregions were evaluated using the Intergroup Correlation Coefficient (ICC), and the ICCs of the features finally selected by each model were all greater than 0.85 after the robustness assessment.

**Table 2:** Radiomic feature selection results of the prediction model.

Model	Model 1	Model 2 (T1)	Model 3 (T2)	Model 4 (FLAIR)	Model 5 (T1C)
Number of selected features	7	9	4	5	6
Individual features	T1rCET-LargeAreaEmphasis	T1rCET-LargeAreaEmphasis	T2rCET-GLCMEntropy_angle45_offset1	FLAIRrCET-HaralickCorrelation_angle135_offset7	T1CrCET-ShortRunHighGreyLevelEmphasis_AllDirection_offset7_SD
	T1rNec-LargeAreaEmphasis	T1rCET-GLCMEntropy_angle90_offset4	T2rCET-GLCMEntropy_angle90_offset7	FLAIRrCET-InverseDifferenceMoment_angle135_offset4	T1CrNec-GLCMEntropy_angle90_offset7
	T2rCET-GLCMEntropy_angle45_offset1	T1rCET-GLCMEntropy_AllDirection_offset1	T2rCET-HaralickCorrelation_angle0_offset7	FLAIRrCET-ShortRunLowGreyLevelEmphasis_angle45_offset4	T1CrNec-ShortRunLowGreyLevelEmphasis_angle0_offset4
	FLAIRrCET-InverseDifferenceMoment_angle135_offset4	T1rCET-InverseDifferenceMoment_AllDirection_offset7_SD	T2rCET-ShortRunLowGreyLevelEmphasis_angle0_offset1	FLAIRrCET-GLCMEntropy_angle90_offset4	T1CrNec-LowGreyLevelRunEmphasis_AllDirection_offset4_SD
	FLAIRrCET-HaralickCorrelation_angle135_offset7	T1rE-ShortRunLowGreyLevelEmphasis_angle45_offset4		FLAIRrCET-InverseDifferenceMoment_angle135_offset7	T1CrNec-GLCMEntropy_AllDirection_offset4_SD
	FLAIRrCET-ShortRunLowGreyLevelEmphasis_angle45_offset4	T1rNec-LargeAreaEmphasis			T1CrNec-InverseDifferenceMoment_AllDirection_offset7_SD
	T1CrNec-ShortRunLowGreyLevelEmphasis_angle0_offset4	T1rNec-Inertia_angle45_offset4			
		T1rNec-GLCMEnergy_AllDirection_offset7_SD			
		T1rNec-MinIntensity			
The best-performance feature	FLAIRrCET-InverseDifferenceMoment_angle135_offset4	T1rCET-GLCMEntropy_angle90_offset4	T2rCET-HaralickCorrelation_angle0_offset7	FLAIRrCET-InverseDifferenceMoment_angle135_offset4	T1CrNec-GLCMEntropy_angle90_offset7
Maximum coefficient	0.899	-0.62	-0.934	0.414	-0.593

Model	Model 6 (rCET)	Model 7 (rE)	Model 8 (rNEC)		
Number of selected features	6	3	5		
Individual features	T1rCET-GLCMEntropy_angle90_offset4	T2rE-ClusterProminence_angle45_offset7	T1rNec-MinIntensity		
	T2rCET-ShortRunLowGreyLevelEmphasis_angle0_offset1	FLAIRrE-GLCMEntropy_angle135_offset7	T1CrNec-ShortRunLowGreyLevelEmphasis_angle0_offset4		
	T2rCET-GLCMEntropy_angle45_offset1	T1CrE-GLCMEntropy_AllDirection_offset1_SD	T1CrNec-GLCMEntropy_angle90_offset7		
	FLAIRrCET-HaralickCorrelation_angle135_offset7		T1CrNec-LowGreyLevelRunEmphasis_AllDirection_offset4_SD		
	FLAIRrCET-ShortRunLowGreyLevelEmphasis_angle45_offset4		T1CrNec-InverseDifferenceMoment_AllDirection_offset7_SD		
	FLAIRrCET-InverseDifferenceMoment_angle135_offset4				
The best-performance feature	FLAIRrCET-InverseDifferenceMoment_angle135_offset4	T2rE-ClusterProminence_angle45_offset7	T1CrNec-GLCMEntropy_angle90_offset7		
Maximum coefficient	0.595	-0.476	-0.48

### Development and performance of predictive models and the nomogram

**Figure 2:**
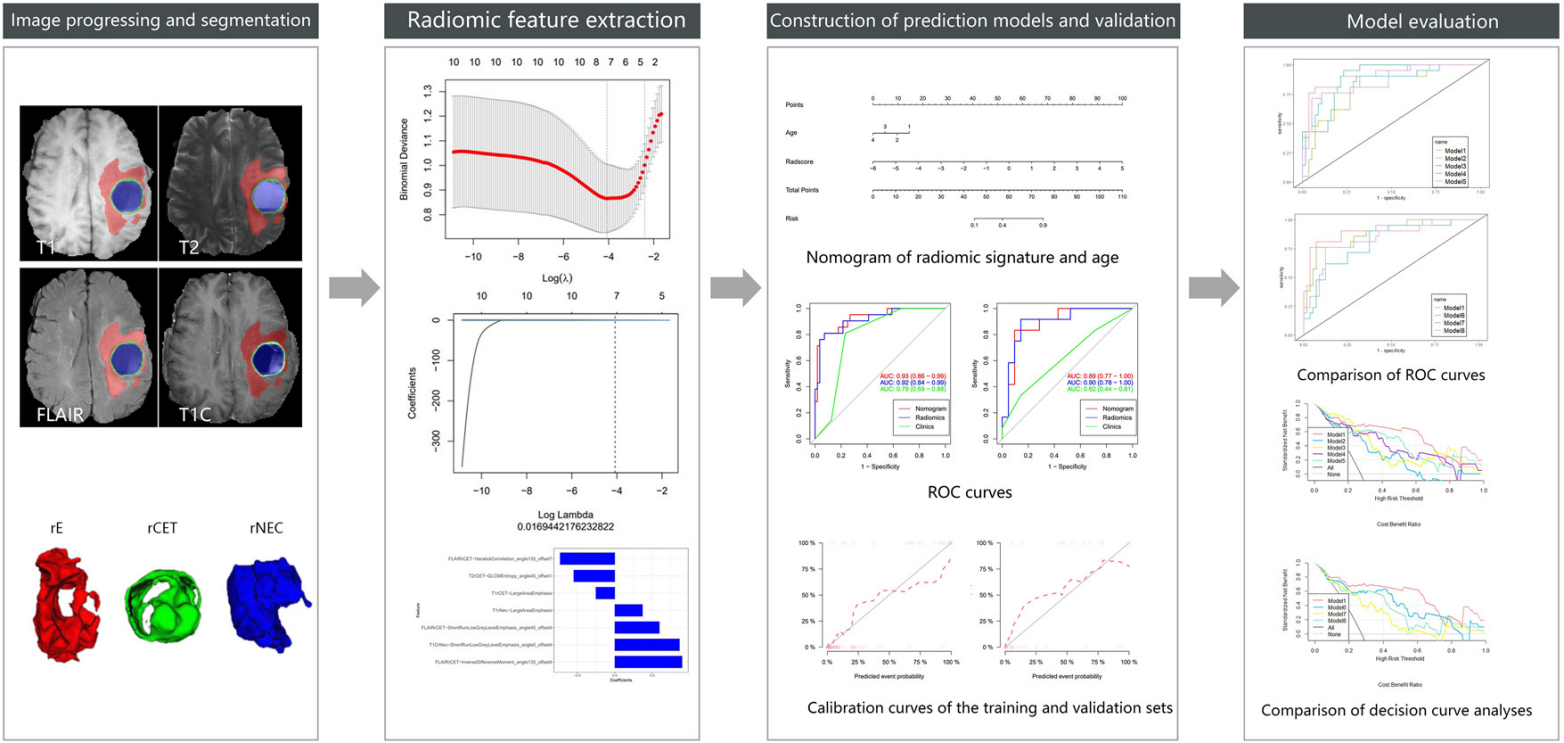
Flowchart of this study.

**Figure 3:**
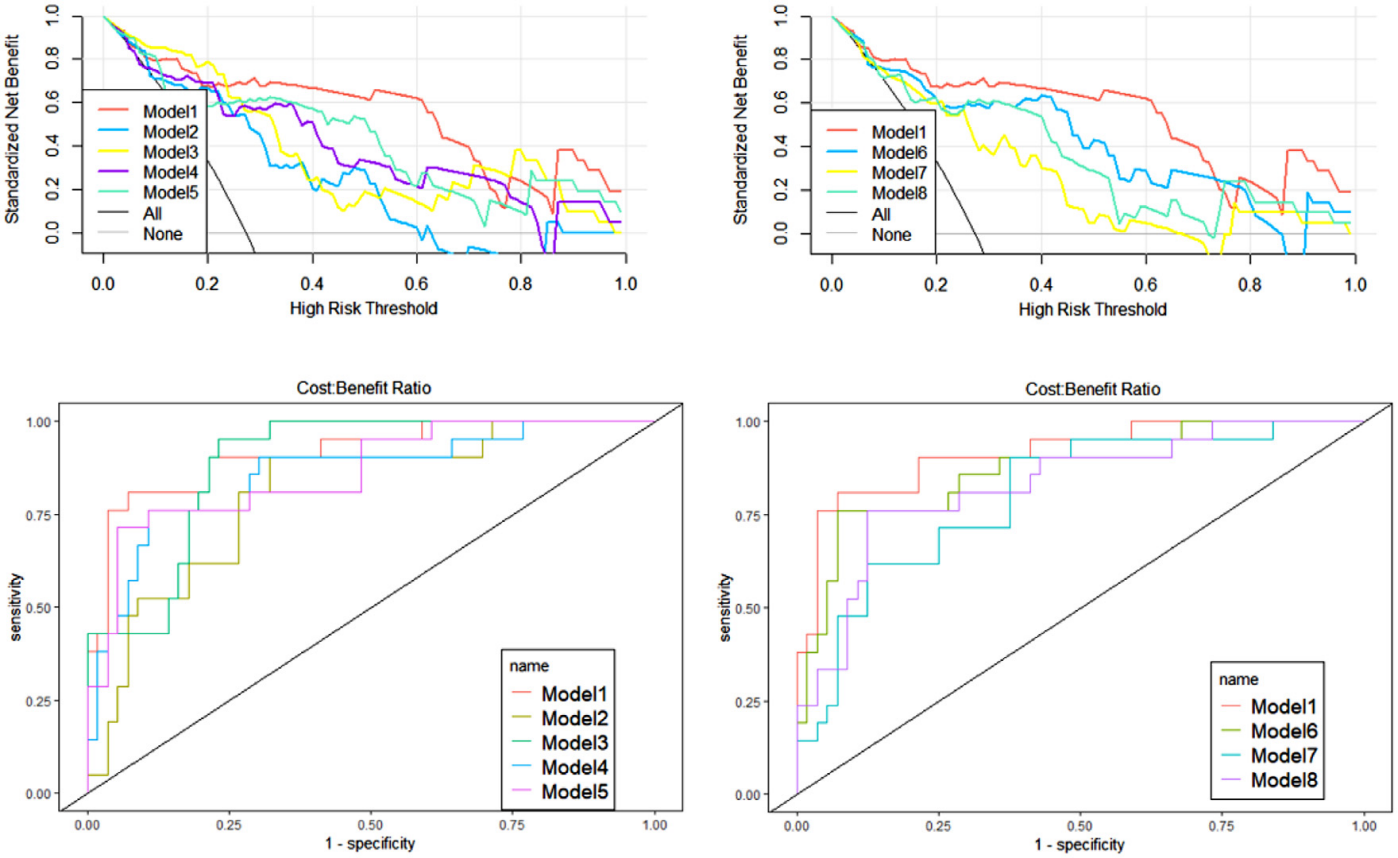
When comparing the eight models, the authors found that Model 1 achieved better performance compared to the other seven models, both in the decision curve and in the ROC curve analysis.

During the construction of the predictive models, logistic regression analysis identified Radscore and age as independent predictors, on the basis of which a visualized nomogram was constructed (Fig. 2). Model 1 reached the highest AUC (training set, 0.93 [95% CI 0.86‒0.99]; validation set, 0.89 [95% CI 0.77‒1.00)]) (Fig. 3), with a sensitivity, specificity, and accuracy of 0.81, 0.93, and 0.90 and 0.77, 0.90, and 0.85 in the training and validation sets, respectively (Table 3). The calibration curves of the nomogram (Fig. 2) showed good agreement between the predictive possibility and observed outcomes of IDH1 mutations in both the training and validation sets (p = 0.297 and 0.148, Hosmer-Lemeshow test). Decision curve analysis showed that the net benefit of the combined model was higher than that of the other models for almost the entire range of Pt-values (Fig. 3). The results and discussion may be presented separately, or in one combined section, and may optionally be divided into headed subsections.

**Table 3:** A performance summary of models.

Model	Train group				Validation group			
	AUC^a^ (95% CI)	Accuracy (%)	Sensitivity (%)	Specificity (%)	AUC (95%CI)	Accuracy (%)	Sensitivity (%)	Specificity (%)
Model 1	0.93 (0.86‒0.99)	89.6	81.0	92.9	0.89 (0.77‒1.00)	84.8	76.9	90.0
Model 2 (T1)	0.87 (0.78‒0.95)	85.7	85.7	85.7	0.78 (0.57‒0.99)	78.8	77.8	79.2
Model 3 (T2)	0.91 (0.84‒0.97)	81.8	100.0	75.0	0.79 (0.59‒0.98)	81.8	75.0	86.0
Model 4 (FLAIR)	0.90 (0.82‒0.98)	88.3	85.7	89.3	0.77 (0.60‒0.93)	69.7	60.0	73.9
Model 5 (T1C)	0.91 (0.84‒0.98)	84.4	95.2	80.4	0.78 (0.57‒0.98)	72.7	60.0	83.3
Model 6 (rCET)	0.90 (0.83‒0.98)	84.4	85.7	83.9	0.79 (0.63‒0.95)	72.7	63.6	77.2
Model 7 (rE)	0.89 (0.80‒0.97)	87.0	81.0	89.3	0.67 (0.47‒0.87)	75.8	83.3	74.0
Model 8 (rNEC)	0.88 (0.80‒0.96)	80.5	95.2	75.0	0.73 (0.53‒0.94)	69.7	57.1	78.9

a AUC, Means Area Under Curve; CI, Means Confidence Interval.

## Discussion

In recent years, in-depth studies on the molecular structure of gliomas have revealed their unique genetic features and epigenetic manifestations, and they have been classified into different molecular subtypes accordingly. Epigenetics is associated with DNA methylation, and the IDH1 gene induces demethylation;^18^ therefore, epigenetic regulation of the IDH1 gene becomes a key biomarker for tumor classification and plays a critical role in the evolution and biological expression of gliomas.^19^ Qi et al.^20^ found that overexpression of the IDH1-R132H mutation in Glioma Stem Cells (GSCs) leads to reduced GSC proliferation, migration, and invasion, induces apoptosis and improves GSC differentiation. Notably, mutations at this locus occur in more than 80% of IDH1-M gliomas; as a result, patients with IDH1-M gliomas generally have a better prognosis.^21^, ^22^, ^23^ Su et al.^10^^,^^14^^,^^16^^,^^24^ found that the mean age of patients with IDH1-M glioma was lower than that of patients with IDH1-W glioma. In the present study, the age feature also showed strong predictive strength, implying that the nomogram incorporating the age feature would have better predictive power.^14^^,^^25^ Accordingly, the present study added age to the column line graph with the expectation of improving its predictive power.

Yu et al.^26^ examined the imaging histology of IDH1 gene mutation in grade II gliomas and included glioma grade as an important feature in the study, constructing an imaging histology model with an AUC value of 0.86. However, the glioma grade needed to be obtained pathologically after surgery or biopsy, and because this study was based on non-invasive imaging histology, the glioma grade was not included as a clinical feature.

As mentioned earlier, the authors employed four conventional MRI sequences and out-lined three different regions of interest, and then combined the clinical features with the Radscore signature to build eight separate models. Among the models 2‒5, and 3 and 5 (T2 and T1C sequences) having the highest AUC, the gliomas often had neo-vascularization within them, which can be accompanied locally by blood-brain barrier disruption. This information can usually be shown in the T1C sequence,^14^^,^^27^ which can show both the general border of the tumor and be used to assess the aggressiveness of the tumor. For this reason, in several previous studies, MR images based on T1C sequences were mostly used.^28^, ^29^, ^30^ In contrast, in model 1, three of the seven extracted radiomic features were from FLAIR sequences, and the sum of the absolute values of the feature coefficients reached the highest value of approximately 2.227, followed by T1C sequences at approximately 0.865. The absolute value of the feature coefficient can be regarded as the importance of a feature for predicting the risk of mutations in the IDH1 gene, and the larger the absolute value, the more significant the effect on the classification. In previous studies on glioma,^28^^,^^31^^,^^32^ researchers found that features from FLAIR sequences also showed good performance for glioblastoma. FLAIR and T2 can provide critical information about peritumoral edema, which is considered an essential biological behavior of gliomas, whose main components are tumor cells and branching capillaries, with a higher cell density indicative of a greater malignancy.^33^^,^^34^ These two sequences also showed a strong predictive power in the present research.

Some relevant studies were based on sketching the overall glioma region when constructing radiomic models to predict IDH1 gene mutations.^26^^,^^35^ According to the present study, tumor rCET, rNEC and rE had significant differences in information about cell density, microvascular proliferation, and local microenvironment, and extracting the radiomic features of different ROIs separately can better quantify the comprehensive information of tumors and characterize the heterogeneity of gliomas. In models 6‒8, the highest AUC value was reached in model 6, and the highest characteristic coefficient of CET was matched in model 1. The rCET is the region with the highest tumor cell density, more active growth, and more pronounced heterogeneity, so the contribution of this region to the predictive efficacy of IDH1 gene mutation is greater than that of the other two regions. Most studies on gliomas point to a correlation between enhancing regions and prognosis. For example, Baldock^36^ et al. demonstrated that IDH1-M was more aggressive than IDH1-W in enhancing gliomas and developed a model of tumor growth in order to provide better treatment alternatives. Molinaro^37^ et al. demonstrated the connection between maximal resection of CE tumors and OS in patients with glioblastoma. These findings may help to develop individualized strategies for surgery in patients with glioma.

As mentioned above, different sequences and different regions of interest can provide different information about the tumor interior,^38^, ^39^, ^40^, ^41^ and the combined quantification of this information can achieve better predictive results. Model 1 constructed in this study achieved AUC values of 0.91 and 0.86 in the training and test groups, respectively, with a good fit of the calibration curve (p = 0.751, Hosmer-Lemeshow test), indicating that its predictive ability was better than that of the other models. The seven radiomic features selected in Model 1 included three GLCM features, two GLRLM features, and two GLSZM features, which are frequently used in radiomics and reflect the imaging information of the tumor from the fine and uniform degree of the image to quantitatively describe the internal characteristics of the tumor. The sum of the GLCM feature coefficients was 2.045, which is the statistic describing the grayscale and texture complexity of the image. This feature can reveal a linear correlation between the grayscale values and the respective voxels in the GLCM; the greater the correlation, the more homogeneous the values of the matrix elements. The reason for its higher percentage may be that IDH1-M has a more homogeneous internal structure than IDH1-W, indicating that IDH1-M was less heterogeneous than IDH1-W.^14^ The GLRLM features indicated the roughness of the texture in the preset direction,^16^^,^^42^ and the sum of its feature coefficients was 1.462. The complementarity of multiple features under this type of textural parameter reflected the signal intensity and tumor inhomogeneity in different regions of interest from different gray values and directions, thus achieving a better model estimation effectiveness. The sum of the GLSZM feature coefficients was 0.62. GLSZM was a measure of the distribution of large area size zones, with a greater value indicative of larger size zones and coarser texture.^42^ These radiomic features further improved the predictive performance of model 1, and the decision curve analysis revealed that the net clinical benefit of model 1 was superior to that of the other models over a large range of Pt-values, demonstrating its superiority for clinical application. Accordingly, this comprehensive model based on four conventional sequences and three different ROIs of MR images, combined with clinical features, was able to reflect the heterogeneity of gliomas more comprehensively and completely and may have higher accuracy in predicting IDH1 gene status.

This study has some limitations. First, this was a single-center study, the sample size was not large enough, and the efficiency and stability of the model still need to be improved; the next step will be to increase the sample size to continue the study. In addition, the overlap of various tissue types cannot be avoided when outlining ROIs; for example, some tumor tissues without enhancement may overlap with edema tissues, which may have an impact on the results. Furthermore, the characterization and measurement of MR image-related features depend on the observer's experience, which limits the accuracy and reproducibility of the results.

## Conclusions

In conclusion, a nomogram based on multiparametric, and multiregional MR images did provide better prediction of IDH1 gene mutations. rCET showed better efficacy in these three regions of interest, while the FLAIR sequence was comparable to the T1C sequence in four different sequences, for which more validation should be performed.

## Funding

This research was funded by Medical Health Science and Technology Project of Zhejiang Province, China (Grant nº 2023KY1047), Medical health Science and Technology Project of Zhejiang Province, China (Grant nº 2017KY572), and Ningbo Science and Technology Benefit Project, Zhejiang Province, China (Grant nº 2016C51017).

## Data availability

The data supporting the results of this study are available from the corresponding author (Haibo Dong) upon request.

## Declaration of Competing Interest

The authors declare no conflicts of interest.

## References

[1] Gritsch S, Batchelor TT, Gonzalez Castro LN (2022). Diagnostic, therapeutic, and prognostic implications of the 2021 World Health Organization classification of tumors of the central nervous system. Cancer.

[2] Kurokawa R, Kurokawa M, Baba A, Ota Y, Pinarbasi E, Camelo-Piragua S (2022). Major changes in 2021 world health organization classification of central nervous system tumors. Radiographics.

[3] Zhang C, Moore LM, Li X, Yung WK, Zhang W. (2013). IDH1/2 mutations target a key hallmark of cancer by deregulating cellular metabolism in glioma. Neuro Oncol.

[4] Huang LE. (2019). Friend or foe-IDH1 mutations in glioma 10 years on. Carcinogenesis.

[5] Philip B, Yu DX, Silvis MR, Shin CH, Robinson JP, Robinson GL (2018). Mutant IDH1 promotes glioma formation in vivo. Cell Rep.

[6] Zhang D, Zhu W, Guo J, Chen W, Gu X. (2022). Application of artificial intelligence in glioma researches: a bibliometric analysis. Front Oncol.

[7] Taha B, Boley D, Sun J, Chen CC. (2021). State of Radiomics in Glioblastoma. Neurosurgery.

[8] Zacharaki EI, Wang S, Chawla S, Yoo DS, Wolf R, Melhem ER (2009). Classification of brain tumor type and grade using MRI texture and shape in a machine learning scheme. Magn Reson Med.

[9] Liu Z, Wang S, Dong D, Wei J, Fang C, Zhou X (2019). The applications of radiomics in precision diagnosis and treatment of oncology: opportunities and challenges. Theranostics.

[10] Wang J, Zheng X, Zhang J, Xue H, Wang L, Jing R (2021). An MRI-based radiomics signature as a pretreatment noninvasive predictor of overall survival and chemotherapeutic benefits in lower-grade gliomas. Eur Radiol.

[11] Niu L, Feng WH, Duan CF, Liu YC, Liu JH, Liu XJ. (2020). The value of enhanced MR radiomics in estimating the IDH1 gen-otype in high-grade gliomas. Biomed Res Int.

[12] Zhou H, Xu R, Mei H, Zhang L, Yu Q, Liu R (2022). Application of Enhanced T1WI of MRI Radiomics in Glioma Grading. Int J Clin Pract.

[13] Wang Q, Li Q, Mi R, Ye H, Zhang H, Chen B (2019). Radiomics nomogram building from multiparametric mri to predict grade in patients with glioma: a cohort study. J Magn Reson Imaging.

[14] Tan Y, Zhang S-T, Wei J-W, Dong D, Wang X-C, Yang G-Q (2019). A radiomics nomogram may improve the prediction of IDH genotype for astrocytoma before surgery. Eur Radiol.

[15] Zhang X, Lu H, Tian Q, Feng N, Yin L, Xu X (2019). A radiomics nomogram based on multiparametric MRI might stratify glioblastoma patients according to survival. Eur Radiol.

[16] Su X, Sun H, Chen N, Roberts N, Yang X, Wang W (2020). A radiomics-clinical nomogram for preoperative prediction of IDH1 mutation in primary glioblastoma multiforme. Clin Radiol.

[17] Wu S, Zhang X, Rui W, Sheng Y, Yu Y, Zhang Y (2022). A nomogram strategy for identifying the subclassification of IDH mutation and ATRX ex-pression loss in lower-grade gliomas. Eur Radiol.

[18] Rohle D, Popovici-Muller J, Palaskas N, Turcan S, Grommes C, Campos C (2013). An inhibitor of mutant IDH1 delays growth and promotes differentiation of glioma cells. Science.

[19] Yamashita AS, da Costa Rosa M, Borodovsky A, Festuccia WT, Chan T, Riggins GJ (2019). Demethylation and epigenetic modi-fication with 5-azacytidine reduces IDH1 mutant glioma growth in combination with temozolomide. Neuro Oncol.

[20] Yao Q, Cai G, Yu Q, Shen J, Gu Z, Chen J (2018). IDH1 mutation diminishes aggressive phenotype in glioma stem cells. Int J Oncol.

[21] Platten M, Bunse L, Wick A, Bunse T, Le Cornet L, Harting I (2021). A vaccine targeting mutant IDH1 in newly diagnosed glioma. Nature.

[22] Rossetto M, Ciccarino P, Boisselier B, Labussiere M, Sanson M. (2011). Metabolism of glioma and IDH1/IDH2 mutations. Rev Neurol (Paris).

[23] Yan H, Parsons DW, Jin G, McLendon R, Rasheed BA, Yuan W (2009). IDH1 and IDH2 mutations in gliomas. N Engl J Med.

[24] Tian H, Wu H, Wu G, Xu G. (2020). Noninvasive prediction of TERT promoter mutations in high-grade glioma by radiomics analysis based on multiparameter MRI. Biomed Res Int.

[25] Lao J, Chen Y, Li Z-C, Li Q, Zhang J, Liu J (2017). A deep learning-based radiomics model for prediction of survival in glioblastoma multi-forme. Sci Rep.

[26] Yu J, Shi Z, Lian Y, Li Z, Liu T, Gao Y (2017). Noninvasive IDH1 mutation estimation based on a quantitative radiomics approach for grade II glioma. Eur Radiol.

[27] Santarosa C, Castellano A, Conte GM, Cadioli M, Iadanza A, Terreni MR (2016). Dynamic contrast-enhanced and dynamic susceptibility contrast perfusion MR imaging for glioma grading: Preliminary comparison of vessel compartment and permeability parameters using hotspot and histogram analysis. Eur J Radiol.

[28] Boxerman JL, Zhang Z, Safriel Y, Rogg JM, Wolf RL, Mohan S (2018). Prognostic value of contrast enhancement and FLAIR for survival in newly diag-nosed glioblastoma treated with and without bevacizumab: results from ACRIN 6686. Neuro Oncol.

[29] Wang J, Hu Y, Zhou X, Bao S, Chen Y, Ge M (2022). A radiomics model based on DCE-MRI and DWI may improve the prediction of estimating IDH1 mutation and angiogenesis in gliomas. Eur J Radiol.

[30] Ding J, Zhao R, Qiu Q, Chen J, Duan J, Cao X (2022). Developing and validating a deep learning and radiomic model for glioma grading using multiplanar reconstructed magnetic resonance contrast-enhanced T1-weighted imaging: a robust, multi-institutional study. Quant Imaging Med Surg.

[31] Li J, Liu S, Qin Y, Zhang Y, Wang N, Liu H. (2020). High-order radiomics features based on T2 FLAIR MRI predict multiple glioma immunohistochemical features: a more precise and personalized gliomas management. PLoS One.

[32] Prasanna P, Patel J, Partovi S, Madabhushi A, Tiwari P. (2017). Radiomic features from the peritumoral brain parenchyma on treatment-naïve multi-parametric MR imaging predict long versus short-term survival in glioblastoma multiforme: pre-liminary findings [published correction appears in Eur Radiol. 2017 Jun 12]. Eur Radiol.

[33] Wang X, Liu X, Chen Y, Lin G, Mei W, Chen J (2015). Histopathological findings in the peritumoral edema area of human glioma. Histol Histopathol.

[34] Li ZC, Bai H, Sun Q, Zhao Y, Lv Y, Zhou J (2018). Multiregional radiomics profiling from multiparametric MRI: identifying an imaging predictor of IDH1 mutation status in glioblastoma. Cancer Med.

[35] Hsieh KL, Chen CY, Lo CM. (2017). Radiomic model for predicting mutations in the isocitrate dehydrogenase gene in glioblas-tomas. Oncotarget.

[36] Baldock AL, Yagle K, Born DE, Ahn S, Trister AD, Neal M (2014). Invasion and proliferation kinetics in enhancing gliomas predict IDH1 mutation status. Neuro Oncol.

[37] Molinaro AM, Hervey-Jumper S, Morshed RA, Young J, Han SJ, Chunduru P (2020). Association of maximal extent of resection of contrast-enhanced and non-contrast-enhanced tumor with survival within molecular subgroups of patients with newly diagnosed glioblastoma [published correction appears in JAMA Oncol. 2020;6(3):444]. JAMA Oncol.

[38] Kobayashi K, Miyake M, Takahashi M, Hamamoto R. (2021). Observing deep radiomics for the classification of glioma grades. Sci Rep.

[39] Xu Y, He X, Li Y, Pang P, Shu Z, Gong X. (2021). The Nomogram of MRI-based radiomics with complementary visual features by machine learning improves stratification of glioblastoma patients: a multicenter study. J Magn Reson Imaging.

[40] Yan J, Zhang B, Zhang S, Cheng J, Liu X, Wang W (2021). Quantitative MRI-based radiomics for noninvasively predicting molecular subtypes and survival in glioma patients. NPJ Precis Oncol.

[41] Lam LHT, Do DT, Diep DTN, Nguyet DLN, Truong QD, Tri TT (2022). Molecular subtype classification of low-grade gliomas using magnetic resonance imaging-based radiomics and machine learning. NMR Biomed.

[42] van Griethuysen JJM, Fedorov A, Parmar C, Hosny A, Aucoin N, Narayan V (2017). Computational radiomics system to decode the radiographic phenotype. Cancer Res.

